# Crystal structure of 5-[bis­(methyl­sulfon­yl)meth­yl]-1,3-dimethyl-5-(methyl­sulfon­yl)pyrimidine-2,4,6(1*H*,3*H*,5*H*)-trione

**DOI:** 10.1107/S2056989014027455

**Published:** 2015-01-01

**Authors:** Eyad Mallah, Ahmed Al-Sheikh, Kamal Sweidan, Wael Abu Dayyih, Manfred Steimann

**Affiliations:** a Faculty of Pharmacy and Medical Science, University of Petra, Amman, Jordan; b Department of Chemistry, Faculty of Science, University of Jordan, Amman, Jordan; c Institut für Anorganische Chemie der Universität Tübingen, Auf der Morgenstelle 18, 72076 Tübingen, Germany

**Keywords:** crystal structure, barbituric acid, pyrimidines, methyl­sulfon­yl, trione, hydrogen bonding

## Abstract

In the title compound, C_10_H_16_N_2_O_9_S_3_, the pyrimidine ring of the 1,3-dimethyl barbituric acid moiety has an envelope conformation with the C atom carrying the methyl­sulfonyl and bis­(methyl­sulfon­yl)methyl substituents as the flap. The dihedral angle between mean plane of the pyrimidine ring and the S/C/S plane is 72.4 (3)°. In the crystal, mol­ecules are linked *via* C—H⋯O hydrogen bonds, forming a three-dimensional structure.

## Related literature   

For examples of the biological activity of pyrimidines, see: Habibi & Tarameshloo (2011 ▸); Holtkamp & Meierkord (2007 ▸). For aspects of nucleic acid binding, see: Demeunynck *et al.* (2004 ▸). For drug applications of C5-substituted barbituric and 2-thio­barbituric acids, see: Getova & Georgiev (1989 ▸); Kratt *et al.* (1990 ▸); Kotha *et al.* (2005 ▸). For the structures of similar compounds, see: Huang & Chen (1986 ▸); Ye *et al.* (1989 ▸); Al-Sheikh *et al.* (2009 ▸); Awad *et al.* (2014 ▸); Glidewell *et al.* (1995 ▸). For the synthesis of the starting material, see: Sweidan *et al.* (2009 ▸).
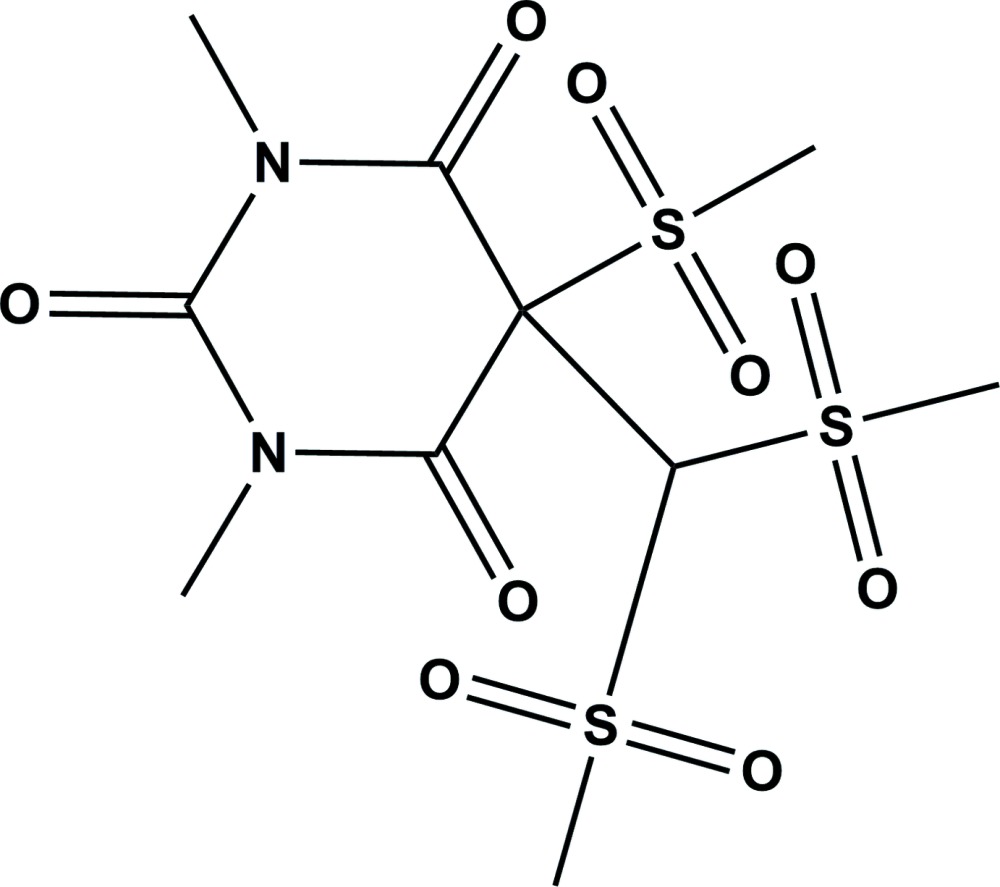


## Experimental   

### Crystal data  

C_10_H_16_N_2_O_9_S_3_
*M* *_r_* = 404.43
Triclinic, 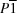

*a* = 7.9415 (16) Å
*b* = 8.5796 (17) Å
*c* = 12.756 (3) Å
α = 77.08 (3)°
β = 79.50 (3)°
γ = 67.83 (3)°
*V* = 779.9 (3) Å^3^
*Z* = 2
Mo *K*α radiation
μ = 0.53 mm^−1^
*T* = 173 K
0.15 × 0.10 × 0.05 mm

### Data collection  

Stoe IPDS diffractometer
11105 measured reflections
3175 independent reflections
2582 reflections with *I* > 2σ(*I*)
*R*_int_ = 0.069

### Refinement  

*R*[*F* ^2^ > 2σ(*F* ^2^)] = 0.060
*wR*(*F* ^2^) = 0.105
*S* = 1.24
3175 reflections
223 parameters
H-atom parameters constrained
Δρ_max_ = 0.39 e Å^−3^
Δρ_min_ = −0.44 e Å^−3^

### 

Data collection: *X-AREA* (Stoe & Cie, 2008 ▸); cell refinement: *X-AREA*; data reduction: *X-RED32* (Stoe & Cie, 2008 ▸); program(s) used to solve structure: *SHELXS97* (Sheldrick, 2008 ▸); program(s) used to refine structure: *SHELXL97* (Sheldrick, 2008 ▸; molecular graphics: *Mercury* (Macrae *et al.*, 2008 ▸); software used to prepare material for publication: *SHELXL97* and *PLATON* (Spek, 2009 ▸).

## Supplementary Material

Crystal structure: contains datablock(s) I, New_Global_Publ_Block. DOI: 10.1107/S2056989014027455/su5040sup1.cif

Structure factors: contains datablock(s) I. DOI: 10.1107/S2056989014027455/su5040Isup2.hkl

Supporting information file. DOI: 10.1107/S2056989014027455/su5040Isup3.cml

**Fig. 1.**
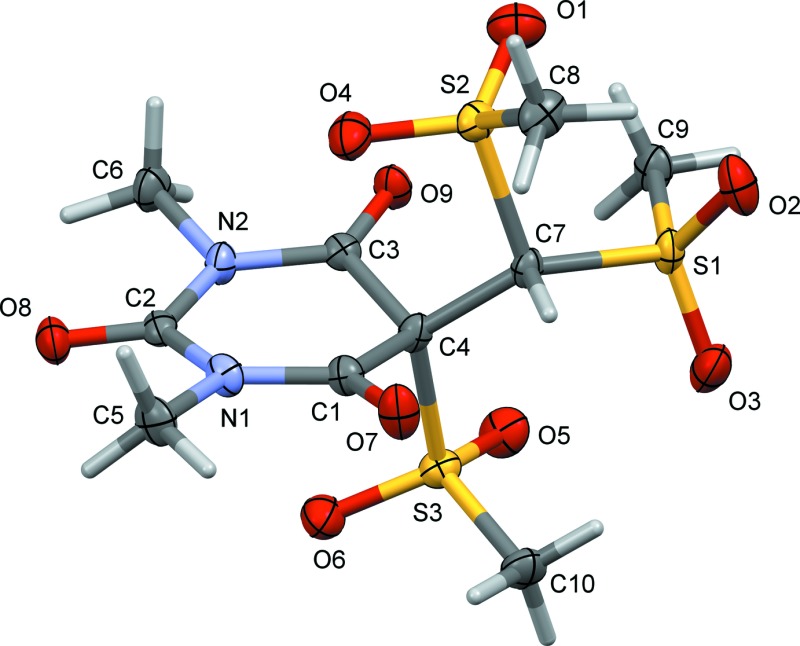
The molecular structure of the title compound, with atom labelling. Displacement ellipsoids are drawn at the 20% probability level.

. DOI: 10.1107/S2056989014027455/su5040fig1.tif
The mol­ecular structure of the title compound, with atom labelling. Displacement ellipsoids are drawn at the 20% probability level.

**Fig. 2.**
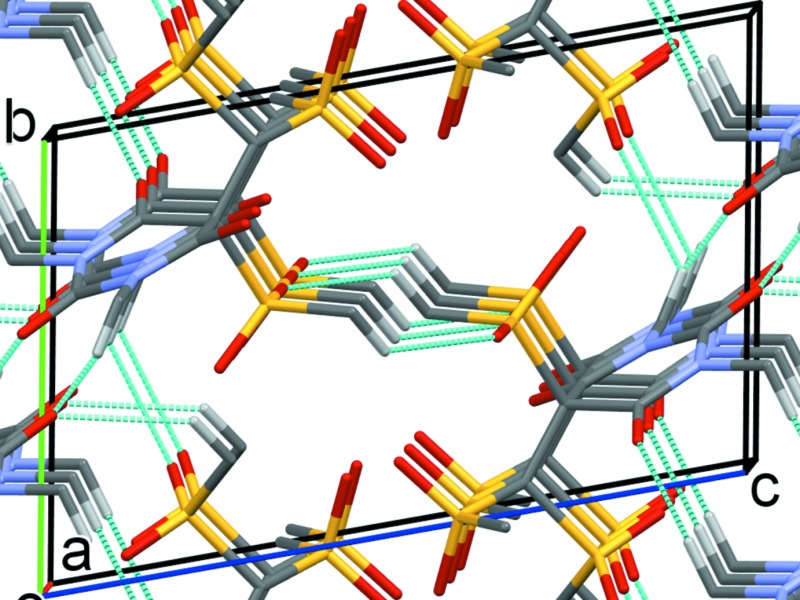
A view along the a axis of the crystal packing of the title compound. The hydrogen bonds are shown as dashed lines (see Table 1 for details).

. DOI: 10.1107/S2056989014027455/su5040fig2.tif
A view along the a axis of the crystal packing of the title compound. The hydrogen bonds are shown as dashed lines (see Table 1 for details).

CCDC reference: 1039815

Additional supporting information:  crystallographic information; 3D view; checkCIF report

## Figures and Tables

**Table 1:** Hydrogen-bond geometry (, )

*D*H*A*	*D*H	H*A*	*D* *A*	*D*H*A*
C5H5*B*O8^i^	0.98	2.51	3.240(6)	131
C6H6*A*O9^ii^	0.98	2.59	3.380(5)	138
C8H8*C*O8^iii^	0.98	2.51	3.263(5)	133
C10H10*A*O5^iv^	0.98	2.50	3.219(5)	130

Symmetry codes: (i) 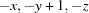; (ii) 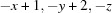; (iii) 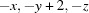; (iv) 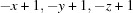.

## supplementary crystallographic information

### S1. Comment

Compounds containing pyrimidine play a vital role in biological activity (Habibi & Tarameshloo, 2011; Holtkamp & Meierkord, 2007). This activity differs from molecule to molecule depending on the tautomery and the nature of the substituents (Demeunynck *et al.*, 2004). In view of the pharmaceutical significance of pyrimidines we became interested in obtaining new barbituric acid derivatives. C5-substituted barbituric and 2-thiobarbituric acids have been used for sedative, hypnotics and anticonvulsant drug applications (Getova & Georgiev 1989; Kratt *et al.*, 1990; Kotha *et al.*, 2005). 1,3-dimethyl barbituric acid has a tendency to accept negative charges by delocalization of π electrons and can exhibit zwitterionic nature. The title compound, which was synthesized *via* the reaction of 1,3-dimethyl-5-bis-(thiomethyl)methylenebarbituric acid and *m*-chloroperbenzoic acid, may find applications in bio-organic chemistry.

Several noteworthy features are evident in the crystal structure of the title compound, Fig. 1. The three central sulfonyl groups utilize one methyl group which have almost identical S—C bond lengths [S1—C9 1.752 (4) Å, S2—C8 1.744 (4) Å, S3—C10 1.762 (4) Å] and they are slightly shorter than those reported for (PhSO_2_)_2_CH_2_ (1.786 Å; Glidewell *et al.* 1995) and for bis(methylsulfonyl)methane (1.781 Å; Awad *et al.* 2014). Interestingly, significant elongation of the (C4—C7) bond length [1.547 (5) Å] may be attributed to interactions between the sulfonyl groups located at position C7.

As a result of crystal packing the geometry of one of the sulfonyl groups is as expected; having one sulfur-oxygen bond slightly shorter than the other [S2—O1 = 1.415 (3) Å, and S1—O3 = 1.434 (3) Å]. The carbonyl groups that are located on the barbituric acid ring have approximately the same bond lengths, varying from 1.200 (4) to 1.211 (4) Å. The pyrimidine ring in barbituric acid moiety is significantly distorted from planarity and has an envelope conformation with atom C as the flap.

In the crystal, molecules are linked via C—H···O hydrogen bonds forming a three-dimensional structure (Table 1 and Fig. 2)

### S2. Experimental

The title compound was synthesized by adding *m*-chloroperbenzoic acid (4.5 g, 20 mmol) to a solution containing 1,3-dimethyl-5-bis-(thiomethyl)methylenebarbituric acid (1.3 g, 5 mmol; Sweidan *et al.*, 2009), in dichloromethane (20 ml) at 213 K. After stirring overnight, the solvent was removed in *vacuo*. Diethylether (20 ml) was added and the precipitated solid was collected and recrystallized from CH_2_Cl_2_/Et_2_O to give colourless crystals (yield: 1.0 g, 51%).

### S3. Refinement

The C-bound H atoms were included in calculated positions and treated as riding atoms: C—H = 0.98–1.00 Å with *U*_iso_(H) = 1.5*U*_eq_(C) for methyl H atoms and = 1.2*U*_eq_(C) for other H atoms.

### Crystal data

**Table:**

C_10_H_16_N_2_O_9_S_3_	*Z* = 2
*M**_r_* = 404.43	*F*(000) = 420
Triclinic, *P*1	*D*_x_ = 1.722 Mg m^−^^3^
Hall symbol: -P 1	Mo *K*α radiation, λ = 0.71073 Å
*a* = 7.9415 (16) Å	Cell parameters from 30 reflections
*b* = 8.5796 (17) Å	θ = 10.3–20.1°
*c* = 12.756 (3) Å	µ = 0.53 mm^−^^1^
α = 77.08 (3)°	*T* = 173 K
β = 79.50 (3)°	Plate, colourless
γ = 67.83 (3)°	0.15 × 0.10 × 0.05 mm
*V* = 779.9 (3) Å^3^	

### Data collection

**Table:**

Stoe IPDS diffractometer	2582 reflections with *I* > 2σ(*I*)
Radiation source: fine-focus sealed tube	*R*_int_ = 0.069
Graphite monochromator	θ_max_ = 26.4°, θ_min_ = 3.1°
phi scans	*h* = −9→9
11105 measured reflections	*k* = −10→10
3175 independent reflections	*l* = −15→15

### Refinement

**Table:**

Refinement on *F*^2^	Secondary atom site location: difference Fourier map
Least-squares matrix: full	Hydrogen site location: inferred from neighbouring sites
*R*[*F*^2^ > 2σ(*F*^2^)] = 0.060	H-atom parameters constrained
*wR*(*F*^2^) = 0.105	*w* = 1/[σ^2^(*F*_o_^2^) + (0.*P*)^2^ + 1.8882*P*] where *P* = (*F*_o_^2^ + 2*F*_c_^2^)/3
*S* = 1.24	(Δ/σ)_max_ < 0.001
3175 reflections	Δρ_max_ = 0.39 e Å^−^^3^
223 parameters	Δρ_min_ = −0.44 e Å^−^^3^
0 restraints	Extinction correction: *SHELXL97* (Sheldrick, 2008, Fc^*^=kFc[1+0.001xFc^2^λ^3^/sin(2θ)]^-1/4^
Primary atom site location: structure-invariant direct methods	Extinction coefficient: 0.0053 (11)

### Special details

**Table:**

Geometry. All e.s.d.'s (except the e.s.d. in the dihedral angle between two l.s. planes) are estimated using the full covariance matrix. The cell e.s.d.'s are taken into account individually in the estimation of e.s.d.'s in distances, angles and torsion angles; correlations between e.s.d.'s in cell parameters are only used when they are defined by crystal symmetry. An approximate (isotropic) treatment of cell e.s.d.'s is used for estimating e.s.d.'s involving l.s. planes.
Refinement. Refinement of *F*^2^ against ALL reflections. The weighted *R*-factor *wR* and goodness of fit *S* are based on *F*^2^, conventional *R*-factors *R* are based on *F*, with *F* set to zero for negative *F*^2^. The threshold expression of *F*^2^ > σ(*F*^2^) is used only for calculating *R*-factors(gt) *etc*. and is not relevant to the choice of reflections for refinement. *R*-factors based on *F*^2^ are statistically about twice as large as those based on *F*, and *R*- factors based on ALL data will be even larger.

### Fractional atomic coordinates and isotropic or equivalent isotropic displacement parameters (Å^2^)

**Table:**

	*x*	*y*	*z*	*U*_iso_*/*U*_eq_	
S1	0.26932 (13)	0.98015 (13)	0.40183 (7)	0.0206 (2)	
S2	0.04979 (12)	1.12489 (12)	0.20775 (7)	0.0203 (2)	
S3	0.36913 (12)	0.56897 (12)	0.32733 (8)	0.0203 (2)	
N1	0.0632 (4)	0.6845 (4)	0.1414 (3)	0.0199 (7)	
N2	0.3363 (4)	0.7382 (4)	0.0629 (2)	0.0194 (7)	
O1	0.1593 (4)	1.2283 (4)	0.1838 (3)	0.0357 (7)	
O2	0.1389 (4)	1.1372 (4)	0.4315 (2)	0.0322 (7)	
O3	0.3197 (4)	0.8360 (4)	0.4871 (2)	0.0312 (7)	
O4	0.0233 (4)	1.0569 (4)	0.1216 (2)	0.0308 (7)	
O5	0.5284 (4)	0.5940 (4)	0.3464 (2)	0.0289 (7)	
O6	0.3920 (4)	0.4469 (4)	0.2607 (2)	0.0287 (6)	
O7	−0.0780 (4)	0.7631 (4)	0.3019 (2)	0.0267 (6)	
O8	0.2123 (4)	0.6024 (4)	−0.0170 (2)	0.0255 (6)	
O9	0.4500 (3)	0.8776 (3)	0.1470 (2)	0.0222 (6)	
C1	0.0528 (5)	0.7430 (5)	0.2337 (3)	0.0186 (7)	
C2	0.2063 (5)	0.6684 (5)	0.0579 (3)	0.0177 (7)	
C3	0.3452 (5)	0.8054 (5)	0.1483 (3)	0.0164 (7)	
C4	0.2215 (5)	0.7758 (5)	0.2529 (3)	0.0171 (7)	
C5	−0.0927 (5)	0.6445 (6)	0.1232 (3)	0.0275 (9)	
H5A	−0.1683	0.6306	0.1920	0.041*	
H5B	−0.0473	0.5383	0.0935	0.041*	
H5C	−0.1662	0.7378	0.0721	0.041*	
C6	0.4587 (5)	0.7625 (5)	−0.0362 (3)	0.0257 (9)	
H6A	0.4152	0.8825	−0.0712	0.039*	
H6B	0.4591	0.6901	−0.0859	0.039*	
H6C	0.5830	0.7309	−0.0175	0.039*	
C7	0.1493 (5)	0.9322 (5)	0.3113 (3)	0.0169 (7)	
H7	0.0390	0.9184	0.3587	0.020*	
C8	−0.1646 (5)	1.2318 (5)	0.2710 (3)	0.0272 (9)	
H8A	−0.1492	1.2715	0.3338	0.041*	
H8B	−0.2333	1.1540	0.2949	0.041*	
H8C	−0.2316	1.3301	0.2199	0.041*	
C9	0.4678 (5)	1.0156 (5)	0.3342 (3)	0.0241 (8)	
H9A	0.5235	1.0504	0.3833	0.036*	
H9B	0.4363	1.1060	0.2711	0.036*	
H9C	0.5546	0.9102	0.3105	0.036*	
C10	0.2393 (6)	0.5223 (5)	0.4497 (3)	0.0280 (9)	
H10A	0.3164	0.4225	0.4954	0.042*	
H10B	0.1361	0.4979	0.4342	0.042*	
H10C	0.1930	0.6206	0.4874	0.042*	

### Atomic displacement parameters (Å^2^)

**Table:**

	*U*^11^	*U*^22^	*U*^33^	*U*^12^	*U*^13^	*U*^23^
S1	0.0214 (5)	0.0306 (5)	0.0163 (4)	−0.0142 (4)	0.0000 (3)	−0.0093 (4)
S2	0.0178 (4)	0.0206 (5)	0.0194 (5)	−0.0040 (4)	0.0005 (3)	−0.0038 (4)
S3	0.0173 (4)	0.0210 (5)	0.0207 (5)	−0.0057 (4)	−0.0023 (4)	−0.0016 (4)
N1	0.0153 (15)	0.0221 (17)	0.0245 (17)	−0.0071 (13)	−0.0042 (12)	−0.0058 (13)
N2	0.0209 (16)	0.0235 (17)	0.0146 (15)	−0.0078 (13)	0.0004 (12)	−0.0070 (13)
O1	0.0328 (17)	0.0331 (18)	0.0394 (18)	−0.0133 (14)	−0.0022 (14)	−0.0010 (14)
O2	0.0262 (15)	0.0404 (18)	0.0368 (17)	−0.0127 (13)	0.0034 (12)	−0.0236 (14)
O3	0.0381 (17)	0.0426 (18)	0.0188 (14)	−0.0216 (14)	−0.0056 (12)	−0.0014 (12)
O4	0.0357 (16)	0.0259 (16)	0.0254 (15)	−0.0020 (13)	−0.0083 (12)	−0.0056 (12)
O5	0.0196 (14)	0.0361 (17)	0.0314 (16)	−0.0106 (12)	−0.0068 (12)	−0.0019 (13)
O6	0.0330 (16)	0.0234 (15)	0.0272 (15)	−0.0053 (12)	−0.0050 (12)	−0.0059 (12)
O7	0.0186 (13)	0.0409 (18)	0.0274 (15)	−0.0162 (12)	0.0049 (11)	−0.0151 (13)
O8	0.0302 (15)	0.0265 (15)	0.0230 (14)	−0.0091 (12)	−0.0045 (12)	−0.0105 (12)
O9	0.0220 (13)	0.0283 (15)	0.0216 (14)	−0.0147 (12)	0.0020 (11)	−0.0083 (11)
C1	0.0161 (17)	0.0206 (19)	0.0201 (18)	−0.0068 (15)	−0.0023 (14)	−0.0043 (15)
C2	0.0185 (17)	0.0152 (18)	0.0179 (18)	−0.0024 (14)	−0.0067 (14)	−0.0025 (14)
C3	0.0127 (16)	0.0166 (18)	0.0185 (18)	−0.0033 (14)	−0.0016 (13)	−0.0035 (14)
C4	0.0150 (16)	0.0226 (19)	0.0150 (17)	−0.0081 (14)	−0.0004 (13)	−0.0040 (14)
C5	0.025 (2)	0.033 (2)	0.033 (2)	−0.0163 (18)	−0.0071 (17)	−0.0088 (18)
C6	0.029 (2)	0.032 (2)	0.0184 (19)	−0.0129 (18)	0.0041 (16)	−0.0089 (17)
C7	0.0159 (17)	0.023 (2)	0.0136 (17)	−0.0085 (15)	0.0010 (13)	−0.0051 (14)
C8	0.0178 (18)	0.031 (2)	0.026 (2)	−0.0027 (17)	0.0022 (16)	−0.0050 (17)
C9	0.0219 (19)	0.035 (2)	0.023 (2)	−0.0167 (17)	−0.0028 (15)	−0.0069 (17)
C10	0.032 (2)	0.031 (2)	0.022 (2)	−0.0180 (19)	0.0004 (17)	0.0011 (17)

### Geometric parameters (Å, º)

**Table:**

S1—O3	1.434 (3)	O9—C3	1.207 (4)
S1—O2	1.436 (3)	C1—C4	1.539 (5)
S1—C9	1.752 (4)	C3—C4	1.539 (5)
S1—C7	1.823 (4)	C4—C7	1.547 (5)
S2—O1	1.415 (3)	C5—H5A	0.9800
S2—O4	1.434 (3)	C5—H5B	0.9800
S2—C8	1.744 (4)	C5—H5C	0.9800
S2—C7	1.876 (4)	C6—H6A	0.9800
S3—O5	1.430 (3)	C6—H6B	0.9800
S3—O6	1.432 (3)	C6—H6C	0.9800
S3—C10	1.762 (4)	C7—H7	1.0000
S3—C4	1.873 (4)	C8—H8A	0.9800
N1—C1	1.358 (5)	C8—H8B	0.9800
N1—C2	1.396 (5)	C8—H8C	0.9800
N1—C5	1.471 (5)	C9—H9A	0.9800
N2—C3	1.365 (5)	C9—H9B	0.9800
N2—C2	1.392 (5)	C9—H9C	0.9800
N2—C6	1.474 (5)	C10—H10A	0.9800
O7—C1	1.211 (4)	C10—H10B	0.9800
O8—C2	1.200 (4)	C10—H10C	0.9800
			
O3—S1—O2	117.09 (19)	C7—C4—S3	117.1 (2)
O3—S1—C9	108.84 (19)	N1—C5—H5A	109.5
O2—S1—C9	109.23 (19)	N1—C5—H5B	109.5
O3—S1—C7	107.80 (17)	H5A—C5—H5B	109.5
O2—S1—C7	102.30 (17)	N1—C5—H5C	109.5
C9—S1—C7	111.45 (17)	H5A—C5—H5C	109.5
O1—S2—O4	118.50 (19)	H5B—C5—H5C	109.5
O1—S2—C8	110.4 (2)	N2—C6—H6A	109.5
O4—S2—C8	108.3 (2)	N2—C6—H6B	109.5
O1—S2—C7	110.32 (17)	H6A—C6—H6B	109.5
O4—S2—C7	104.43 (17)	N2—C6—H6C	109.5
C8—S2—C7	103.80 (18)	H6A—C6—H6C	109.5
O5—S3—O6	118.38 (18)	H6B—C6—H6C	109.5
O5—S3—C10	111.37 (19)	C4—C7—S1	126.6 (3)
O6—S3—C10	108.29 (19)	C4—C7—S2	106.8 (2)
O5—S3—C4	107.38 (17)	S1—C7—S2	110.83 (19)
O6—S3—C4	103.90 (17)	C4—C7—H7	103.3
C10—S3—C4	106.69 (18)	S1—C7—H7	103.3
C1—N1—C2	125.2 (3)	S2—C7—H7	103.3
C1—N1—C5	118.2 (3)	S2—C8—H8A	109.5
C2—N1—C5	116.4 (3)	S2—C8—H8B	109.5
C3—N2—C2	124.9 (3)	H8A—C8—H8B	109.5
C3—N2—C6	116.8 (3)	S2—C8—H8C	109.5
C2—N2—C6	117.7 (3)	H8A—C8—H8C	109.5
O7—C1—N1	123.2 (3)	H8B—C8—H8C	109.5
O7—C1—C4	119.7 (3)	S1—C9—H9A	109.5
N1—C1—C4	117.0 (3)	S1—C9—H9B	109.5
O8—C2—N2	121.6 (3)	H9A—C9—H9B	109.5
O8—C2—N1	120.9 (3)	S1—C9—H9C	109.5
N2—C2—N1	117.4 (3)	H9A—C9—H9C	109.5
O9—C3—N2	123.5 (3)	H9B—C9—H9C	109.5
O9—C3—C4	119.5 (3)	S3—C10—H10A	109.5
N2—C3—C4	116.9 (3)	S3—C10—H10B	109.5
C1—C4—C3	113.4 (3)	H10A—C10—H10B	109.5
C1—C4—C7	106.7 (3)	S3—C10—H10C	109.5
C3—C4—C7	111.2 (3)	H10A—C10—H10C	109.5
C1—C4—S3	105.6 (2)	H10B—C10—H10C	109.5
C3—C4—S3	102.9 (2)		
			
C2—N1—C1—O7	176.2 (4)	O5—S3—C4—C1	179.8 (2)
C5—N1—C1—O7	0.3 (6)	O6—S3—C4—C1	53.6 (3)
C2—N1—C1—C4	−6.8 (5)	C10—S3—C4—C1	−60.7 (3)
C5—N1—C1—C4	177.2 (3)	O5—S3—C4—C3	60.6 (3)
C3—N2—C2—O8	−175.6 (4)	O6—S3—C4—C3	−65.6 (3)
C6—N2—C2—O8	12.9 (5)	C10—S3—C4—C3	−179.9 (2)
C3—N2—C2—N1	7.1 (5)	O5—S3—C4—C7	−61.6 (3)
C6—N2—C2—N1	−164.4 (3)	O6—S3—C4—C7	172.2 (3)
C1—N1—C2—O8	173.7 (4)	C10—S3—C4—C7	57.9 (3)
C5—N1—C2—O8	−10.2 (5)	C1—C4—C7—S1	153.1 (3)
C1—N1—C2—N2	−8.9 (5)	C3—C4—C7—S1	−82.7 (4)
C5—N1—C2—N2	167.1 (3)	S3—C4—C7—S1	35.1 (4)
C2—N2—C3—O9	−173.7 (3)	C1—C4—C7—S2	−73.5 (3)
C6—N2—C3—O9	−2.2 (5)	C3—C4—C7—S2	50.7 (3)
C2—N2—C3—C4	10.1 (5)	S3—C4—C7—S2	168.54 (17)
C6—N2—C3—C4	−178.3 (3)	O3—S1—C7—C4	−55.8 (3)
O7—C1—C4—C3	−160.3 (3)	O2—S1—C7—C4	−179.8 (3)
N1—C1—C4—C3	22.6 (5)	C9—S1—C7—C4	63.5 (4)
O7—C1—C4—C7	−37.5 (5)	O3—S1—C7—S2	172.24 (18)
N1—C1—C4—C7	145.4 (3)	O2—S1—C7—S2	48.2 (2)
O7—C1—C4—S3	87.7 (4)	C9—S1—C7—S2	−68.4 (2)
N1—C1—C4—S3	−89.3 (3)	O1—S2—C7—C4	−110.8 (3)
O9—C3—C4—C1	159.5 (3)	O4—S2—C7—C4	17.5 (3)
N2—C3—C4—C1	−24.2 (5)	C8—S2—C7—C4	130.9 (3)
O9—C3—C4—C7	39.3 (4)	O1—S2—C7—S1	30.6 (2)
N2—C3—C4—C7	−144.4 (3)	O4—S2—C7—S1	158.95 (19)
O9—C3—C4—S3	−86.9 (4)	C8—S2—C7—S1	−87.7 (2)
N2—C3—C4—S3	89.4 (3)		

### Hydrogen-bond geometry (Å, º)

**Table:**

*D*—H···*A*	*D*—H	H···*A*	*D*···*A*	*D*—H···*A*
C5—H5*B*···O8^i^	0.98	2.51	3.240 (6)	131
C6—H6*A*···O9^ii^	0.98	2.59	3.380 (5)	138
C8—H8*C*···O8^iii^	0.98	2.51	3.263 (5)	133
C10—H10*A*···O5^iv^	0.98	2.50	3.219 (5)	130

Symmetry codes: (i) −*x*, −*y*+1, −*z*; (ii) −*x*+1, −*y*+2, −*z*; (iii) −*x*, −*y*+2, −*z*; (iv) −*x*+1, −*y*+1, −*z*+1.
