# Supplementary figures and images for: Crystal structure of 5-[bis­(methyl­sulfon­yl)meth­yl]-1,3-dimethyl-5-(methyl­sulfon­yl)pyrimidine-2,4,6(1H,3H,5H)-trione
Source: Acta Crystallogr E Crystallogr Commun. 2015 Jan 1;71(Pt 1):o58–9. doi: 10.1107/S2056989014027455 (PMC4331903; doi:10.1107/S2056989014027455)

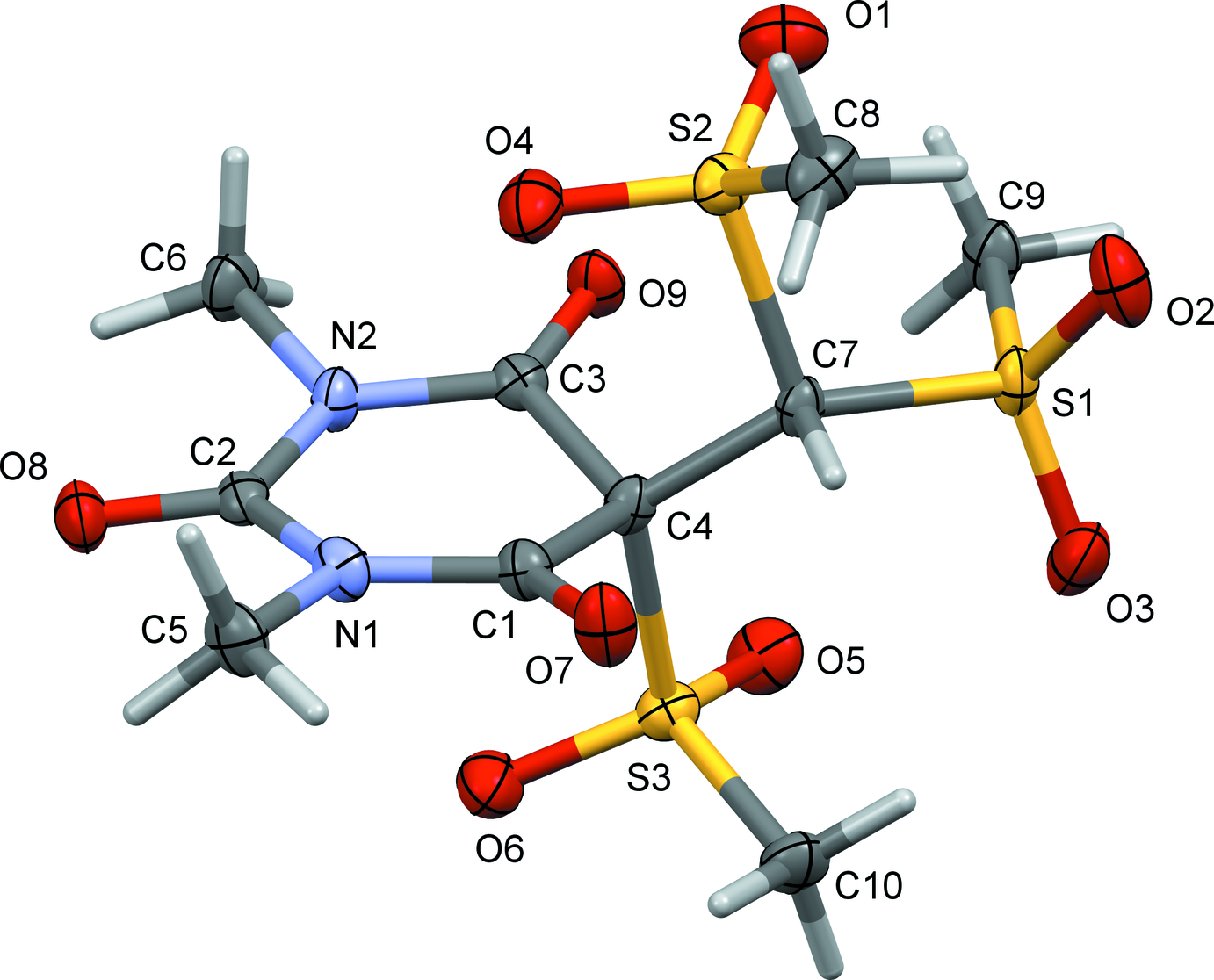

Supplement: Supplementary file 4 [file e-71-00o58-fig1.tif]

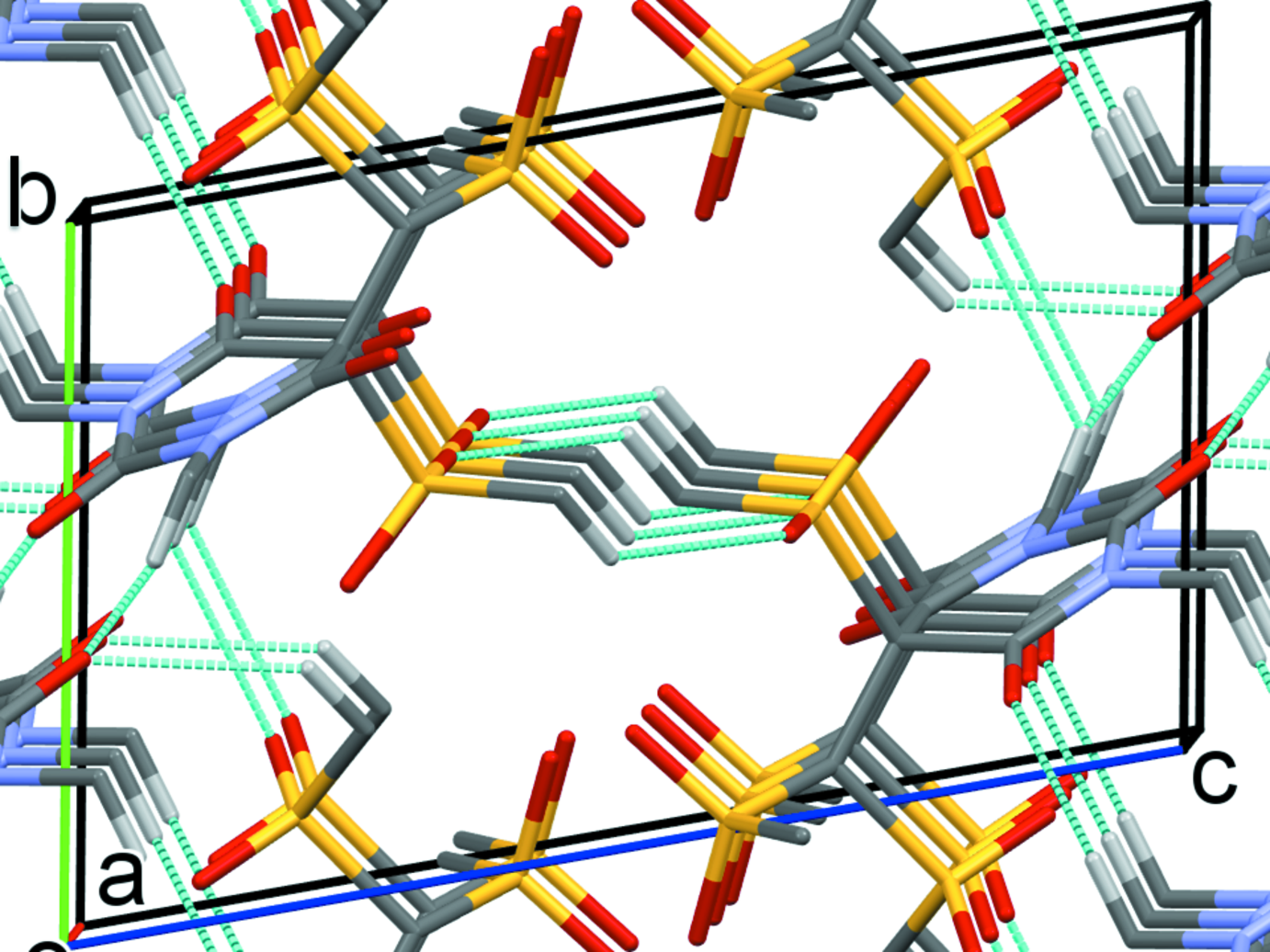

Supplement: Supplementary file 5 [file e-71-00o58-fig2.tif]
